# Effects of Age and Sex on Estimated Diabetes Prevalence Using Different Diagnostic Criteria: The Tromsø OGTT Study

**DOI:** 10.1155/2013/613475

**Published:** 2013-01-09

**Authors:** Moira Strand Hutchinson, Ragnar Martin Joakimsen, Inger Njølstad, Henrik Schirmer, Yngve Figenschau, Johan Svartberg, Rolf Jorde

**Affiliations:** ^1^Tromsø Endocrine Research Group, Department of Clinical Medicine, University of Tromsø, 9037 Tromsø, Norway; ^2^Division of Internal Medicine, University Hospital of North Norway, 9038 Tromsø, Norway; ^3^Department of Community Medicine, University of Tromsø, 9037 Tromsø, Norway; ^4^Department of Medical Biology, University of Tromsø, 9037 Tromsø, Norway; ^5^Division of Laboratory Medicine, University Hospital of North Norway, 9038 Tromsø, Norway

## Abstract

HbA_1c_ 6.5% has recently been recommended as an alternative diagnostic criterion for diabetes. The aims of the study were to evaluate the effects of age, sex, and other factors on prevalence of diabetes and to compare risk profiles of subjects with diabetes when defined by HbA_1c_ and glucose criteria. Subjects were recruited among participants in the longitudinal population-based Tromsø Study. HbA_1c_, fasting plasma glucose, and 2-hour plasma glucose were measured in 3,476 subjects. In total, 294 subjects met one or more of the diagnostic criteria for diabetes; 95 met the HbA_1c_ criterion only, 130 met the glucose criteria only, and 69 met both. Among subjects with diabetes detected by glucose criteria (regardless of HbA_1c_), isolated raised 2-hour plasma glucose was more common in subjects aged ≥ 60 years as compared to younger subjects and in elderly women as compared to elderly men. Subjects with diabetes detected by glucose criteria only had worse cardiometabolic risk profiles than those detected by HbA_1c_ only. In conclusion, the current HbA_1c_ and glucose criteria defined different subjects with diabetes with only modest overlap. Among a substantial proportion of elderly subjects, and especially elderly women, the 2-hour plasma glucose was the only abnormal value.

## 1. Introduction 

Criteria for the diagnosis of diabetes are based on measurements of fasting plasma glucose (FPG), 2-hour plasma glucose (2hPG), or haemoglobin A_1c_ (HbA_1c_). Single raised values with symptoms or raised values on two occasions of any one of these tests, or a combination of these tests can be used for diagnosis of diabetes [1, 2]. The most commonly used test is the FPG as it is simple and inexpensive. The 2hPG is measured in combination with FPG in the oral glucose tolerance test (OGTT), where plasma glucose is measured in the morning after an overnight fast and 2 hours after oral ingestion of 75 g glucose. HbA_1c_ was recently introduced as a diagnostic test for diabetes. Compared to glucose measurements, HbA_1c_ has better sample stability, lower within-person variation and is independent of acute factors such as illness, recent food ingestion, stress, or exercise [3]. 

Diagnostic levels of FPG, 2hPG, and HbA_1c_ are based on thresholds for increased risk of micro- and macrovascular disease, in particular retinopathy [1, 4]. In the DETECT-2 study, sensitivity and specificity for prediction of prevalent retinopathy were almost equal when comparing FPG, 2hPG and HbA_1c_ [5]. Several recent studies have shown that both the prevalence of diabetes and the subjects diagnosed with diabetes vary when different diagnostic criteria for diabetes are applied [6–11]. According to current guidelines, clinicians can choose freely among FPG, OGTT, and HbA_1c_ when testing a patient for diabetes [1, 2]. As HbA_1c_ and glucose criteria have been shown to identify different subjects with diabetes with relatively modest overlap, the choice of test may affect the test outcome [6, 7, 9]. This is important both at the individual level, where correct diagnosis, treatment, and prevention of later complications are in focus, and at the population level where early identification of the “correct” individuals at risk of developing complications is important for cost-effective utilisation of resources. Furthermore, race, age, and sex have been reported to affect the outcome of diabetes testing with different diagnostic criteria [6–8, 12, 13]. This could have implications for the preferred choice of test in subgroups of patients. In Tromsø we have recently performed a large health survey where we measured HbA_1c_, FPG, and 2hPG in 3,476 subjects without previously diagnosed diabetes. These data enabled us to study the effect of age, sex, and other factors on diabetes defined by different diagnostic criteria and to compare cardiometabolic risk profiles of subjects with diabetes defined by different criteria. 

## 2. Materials and Methods 

### 2.1. Subjects

Subjects were recruited from the sixth survey of the longitudinal population-based Tromsø Study performed by the University of Tromsø from October 2007 to December 2008, where HbA_1c_ was measured in 12,769 participants. All subjects without self-reported diabetes and with HbA_1c_ in the range 5.8–6.9% and a random sample of approximately 200 subjects with HbA_1c_ 5.3% and 5.4% and 100 subjects with HbA_1c_ 5.5%, 5.6%, and 5.7%, respectively, were invited to participate in the Tromsø OGTT Study. Race was not registered, but practically all subjects were Caucasian. 

### 2.2. Measurements

Waist and hip circumference, height, weight, and blood pressure were measured, body mass index (BMI) was defined, and physical activity score (PAS) was calculated as previously described [14]. HbA_1c_ was determined by high performance liquid chromatography (HPLC) using an automated analyser (Variant II, Bio-Rad Laboratories Inc., Hercules, CA, USA). The reference interval was 4.3–6.1%. This analysis has been certified by the National Glycohemoglobin Standardization Program (NGSP) as having documented traceability to the Diabetes Control and Complication Trial (DCCT) reference method [15]. Haemoglobin (Hb) was measured by photometry using an automated analyser (reference intervals 11.5–16.0 g/dL for women and 13.0–17.0 g/dL for men). Plasma glucose, serum insulin, and serum C-peptide were measured and analysed as previously described [14]. Serum triglyceride (TG) was analysed with an enzymatic colorimetric assay using an automated clinical chemistry analyser (reference interval 0.5–2.6 mmol/L). Estimates of insulin sensitivity in the fasting state were calculated using homeostasis model assessment (HOMA-IR) and the Quantitative Insulin-Sensitivity Check Index (QUICKI) [16, 17], and insulin sensitivity including the 2-hour values for glucose and insulin with the insulin sensitivity index (ISI_0.120_) according to the formula by Gutt et al. [(*m*/MPG)/log MSI, where *m* = (75 000 mg + [fasting glucose (mg/dL) − 2-h glucose (mg/dL)] × 0.19 × body weight (kg))/120 min, MPG = mean of fasting and 2-h glucose concentrations (mmol/L); MSI = mean of fasting and 2-h insulin concentrations (milliunits per liter)] [18]. 

OGTTs were performed from February 2008 until August 2010 as previously described [14]. All OGTTs were performed in the morning after an overnight fast. To minimize time between OGTT and HbA_1c_, the latter was measured simultaneously with the OGTT from September 2008 onwards. HbA_1c_ from the Tromsø Study 2007-2008 was used for the 932 participants who completed OGTT before September 2008. Mean change in HbA_1c_ for the 2,544 subjects who measured HbA_1c_ on both occasions was −0.03 ± 0.3%. For the purpose of this study, we chose to classify subjects with a single value of FPG ≥ 7.0 mmol/L, 2hPG ≥ 11.1 mmol/L, and/or HbA_1c_ ≥ 6.5% as having diabetes, even though subjects were asymptomatic. Subjects with diabetes were subdivided into diabetes detected by HbA_1c_ only, by OGTT (raised FPG and/or 2hPG) only and by both. Furthermore, subjects with diabetes detected by OGTT (regardless of HbA_1c_) were subdivided into diabetes detected by FPG (regardless of 2hPG) and by isolated raised 2hPG. 

### 2.3. Statistics

Normal distribution was evaluated by visual inspection of histograms and determination of skewness and kurtosis, and after natural log transformation of TG, PAS, QUICKI, HOMA-IR, and ISI_0.120_, all variables except the PAS (where several subjects had “0” values) were considered normally distributed. Ln values were used when these variables were dependent variables. Pearson Chi-square test was used for subgroup analysis in Table 2. Comparisons between groups were performed with logistic regression for categorical variables and univariate analysis of variance with Bonferroni post hoc adjustment or Mann Whitney *U* test for continuous variables in Table 3. Venn diagrams were constructed to illustrate overlap between diagnostic criteria and scatterplots to illustrate the distribution of FPG and 2hPG values in relation to HbA_1c_. Unless otherwise stated, data are expressed as mean ± SD for normally distributed values and as median (5, 95 percentile) for non-normally distributed values. All tests were two-sided, and *P* value < 0.05 was considered statistically significant. The Statistical Package for Social Sciences version 17.0 was used for all statistical analyses (SPSS Inc., Chicago, IL, USA).

## 3. Results

Among the 4,393 subjects who were invited, 3,520 attended and 3,476 completed the OGTT. The number of subjects planned to participate, invited to OGTT, and attended at different HbA_1c_ levels, as measured in the Tromsø Study 2007-2008, is presented in Table 1. In total, 294 (8.5%) subjects met one or more of the diagnostic criteria for diabetes. Mean age was 61 years and 49.5% were women.

### 3.1. Prevalence of Diabetes Defined by Different Diagnostic Criteria

Among those who completed OGTT, 164 (4.7%) met the HbA_1c_ criterion, 119 (3.4%) met the FPG criterion, and 126 (3.6%) met the 2hPG criterion. In total 199 (5.7%) met the OGTT (FPG and/or 2hPG) criteria. As presented in Table 2, 95 (32.3%) of those with diabetes met the HbA_1c_ criterion only, 130 (44.2%) met the OGTT criteria only, and 69 (23.5%) met both criteria. The overlap between subjects with diabetes defined by HbA_1c_ and OGTT varied between 10–35% in different subgroups. 

HbA_1c_ alone detected more subjects with diabetes as compared to OGTT alone in those with BMI < 25 kg/m^2^, TG < 1.2 mmol/L, and high PAS, but there were no significant differences in subgroup analysis of age and sex (Table 2). Among those with diabetes detected by OGTT (regardless of HbA_1c_), isolated raised 2hPG was more common in subjects aged ≥ 60 years and women (Table 2). This effect of age and sex was not due to differences in BMI. Stratification for age showed that the sex difference was significant only in those aged ≥ 60 years, where 58% of women and 36% of men had isolated raised 2hPG (*P* < 0.01). Mean age and BMI did not differ significantly between men and women. Furthermore, the sex difference was significant only in the two lower BMI groups (*P* < 0.05) and in the lowest PAS tertile (*P* < 0.05). 

**Figure 1:**
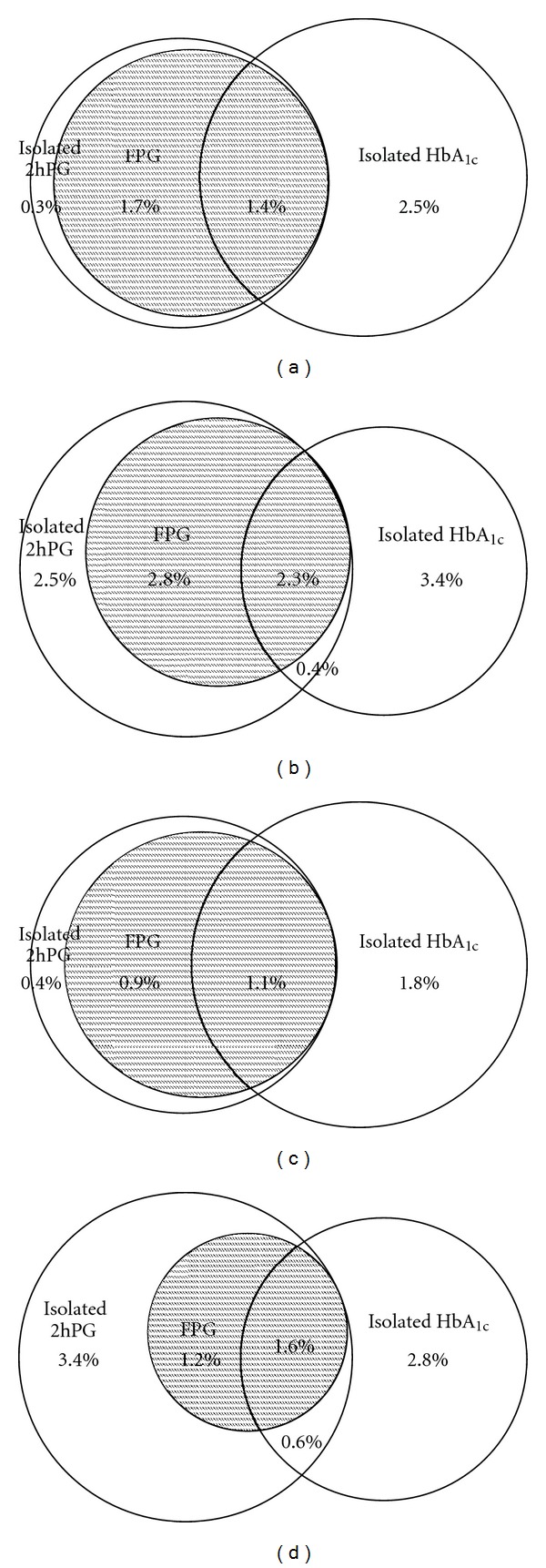
Diabetes prevalence by different diagnostic criteria. Venn diagrams illustrating prevalence of diabetes (%) defined by OGTT criteria (FPG and isolated raised 2hPG) and HbA_1c_ in (a) men aged < 60 years; (b) men aged ≥ 60 years; (c) women aged < 60 years; (d) women ≥ 60 years. The Tromsø OGTT Study.

**Figure 2:**
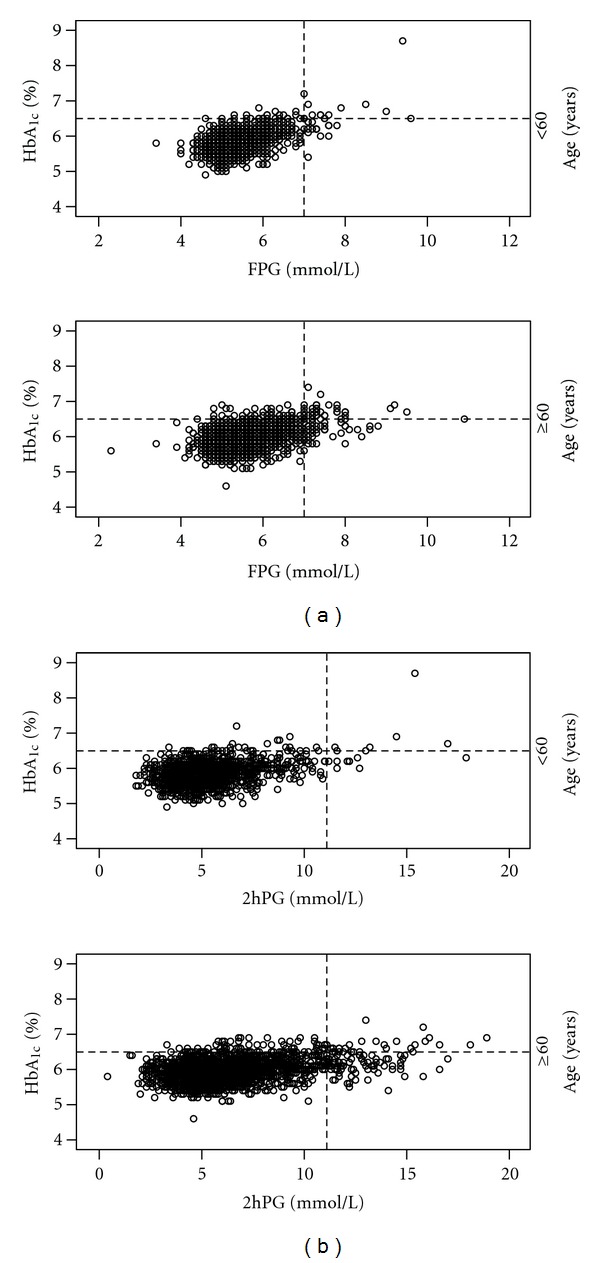
Distribution of FPG and 2hPG values in relation to HbA_1c_. Scatterplots illustrating the distribution of (a) FPG and (b) 2hPG values in relation to HbA_1c_ in subjects aged < 60 years and subjects aged ≥ 60 years. Stippled lines show cut-off points for diabetes. The Tromsø OGTT Study.

The distribution of subjects with diabetes detected by HbA_1c_ only, OGTT only, and both, as well as by OGTT components (FPG and isolated raised 2hPG) is illustrated stratified for age and sex in Figure 1. The overlap between subjects with diabetes defined by HbA_1c_ and OGTT was relatively consistent, but prevalence of isolated raised 2hPG was higher in subjects aged ≥ 60 years as compared to younger subjects, and in elderly women as compared to elderly men. In subjects aged ≥ 60 years the distribution of 2hPG values in relation to HbA_1c_ values was more scattered as compared to younger subjects (Figure 2), illustrating that for many subjects in this age group an HbA_1c_ value < 6.5% did not exclude a 2hPG value above the cut off point for diabetes. 

### 3.2. Characteristics of Subjects with Diabetes Defined by Different Diagnostic Criteria

As presented in Table 3, subjects with diabetes detected by HbA_1c_ only had lower TG, lower systolic blood pressure, higher insulin sensitivity and were less insulin resistant and more physically active as compared to subjects with diabetes detected by OGTT only. Among subjects with diabetes detected by OGTT (regardless of HbA_1c_), those with raised FPG differed from those with isolated raised 2hPG by being younger, predominantly men and more insulin resistant (Table 3). 

## 4. Discussion

### 4.1. Prevalence of Diabetes Defined by Different Diagnostic Criteria

In our population, we found prevalence of diabetes detected by OGTT only to be higher than prevalence of diabetes detected by HbA_1c_ only. The present study also confirmed results from recent studies showing that HbA_1c_ and OGTT define different subjects with diabetes with relatively modest overlap, which in our study was only 23.5% [6, 7, 9]. Prevalence of diabetes defined by HbA_1c_ and OGTT, and overlap between these, differs in previous studies, probably due to differences in age, race, and sex composition of the populations and/or lack of standardisation of HbA_1c_ and glucose measurements [7, 8, 12, 13, 19].

Race, age, and sex have been reported to affect the outcome of diabetes testing with different diagnostic criteria [6–8, 12, 13]. Our study population did not allow us to study the effect of race as practically all subjects were Caucasian. When comparing subjects aged ≥ 60 years with younger subjects, we found no difference in prevalence of diabetes detected by HbA_1c_ only and OGTT only. Among those with diabetes detected by OGTT (regardless of HbA_1c_), prevalence of isolated raised 2hPG was higher in older (≥60 years) as compared to younger subjects. Furthermore, we found that among subjects aged ≥ 60 years, having a 2hPG in the diabetic range but a nondiabetic HbA_1c_ value was more common as compared to younger subjects. Similarly, in the Finnish population-based cross sectional FIN-D2D study including 2,826 men and women aged 45–74 years, any given HbA_1c_ value was found to imply a much higher 2hPG and slightly lower FPG in elderly as compared to middle aged subjects [13]. The 2hPG is known to increase more with age than FPG [20, 21]. Possible explanations for the increased prevalence of isolated raised 2hPG among elderly subjects could be reduced basal insulin secretion [22], delayed insulin response after oral glucose intake [21], physical inactivity, and/or weight gain [23]. 

In our data, there was no sex difference in diabetes detected by HbA_1c_ only and OGTT only. However, we found that among those with diabetes detected by OGTT (regardless of HbA_1c_), isolated raised 2hPG was more common in elderly women as compared to elderly men, a difference that could not be explained by differences in age or BMI. Similarly, the FIN-D2D study reported that HbA_1c_ tends to miss more elderly diabetic people and especially women [13]. Previous studies have suggested that differences in FPG and HbA_1c_ levels are likely to reflect sex-specific differences in glucose regulation as they, unlike differences in 2hPG, remained after adjusting for height and body composition [24, 25]. We also found that HbA_1c_ alone detected more subjects with diabetes as compared to OGTT alone in subjects with BMI < 25 kg/m^2^ as compared to those with higher BMI. In a recently published paper, we reported that a particular HbA_1c_ value implied relatively higher 2hPG and FPG in subjects with high BMI compared to subjects with lower BMI [14]. As very few reports have addressed this issue, it remains uncertain whether BMI has an effect on diagnosis of diabetes by different criteria.

### 4.2. Characteristics of Subjects with Diabetes Defined by Different Diagnostic Criteria

In our population, subjects with diabetes detected by OGTT only had a worse cardiometabolic risk profile than those detected by HbA_1c_ only_._ Previous studies have shown conflicting results; some have found the worst risk profiles in subjects with diabetes defined by OGTT [6, 8, 9], some in subjects with diabetes defined by HbA_1c_ [26], and some have found the two groups to have equally unfavourable risk profiles [8, 10]. In the international A1C-Derived Average Glucose study including 427 subjects with diabetes, HbA_1c_, FPG, and 2hPG were all associated with CVD risk factors, but the strongest association was seen with HbA_1c_ [27]. We did not have data to evaluate the risk of diabetes complications in the different groups. Although both HbA_1c_ and 2hPG have been shown to be independent risk factors for cardiovascular morbidity and mortality, the added prognostic information may be marginal as compared to standard nonglycaemic risk factors [28–30]. In a prospective study based on the Norwegian population-based longitudinal HUNT study, the risk of macrovascular complications in subjects with relatively low HbA_1c_ values was found to be mainly related to conventional risk factors [31]. 

The strength of our study is that OGTT was performed in a large number of subjects recruited from a population representative of the general population in our area. The main shortcomings of our study are that only subjects with HbA_1c_ in the range of 5.3–6.9% were invited to participate and that subjects included at an early stage of the study did not have HbA_1c_ measured simultaneously with the OGTT, but were included in the analysis with the HbA_1c_ value measured in the Tromsø Study 2007-2008. We chose to include these subjects in the analysis as we found that change in HbA_1c_ from the Tromsø Study to the OGTT visit was negligible for those who had HbA_1c_ measured at both occasions. Furthermore, in the absence of clear symptoms, diagnosis of diabetes requires raised values of HbA_1c_, FPG, or 2hPG on two occasions. For practical reasons, we did not repeat either HbA_1c_, or the OGTTs, but chose to classify subjects with a single raised value of HbA_1c_, FPG, or 2hPG as having diabetes. As FPG, and especially 2hPG, are known to have high within-person variation, repeating the OGTTs to confirm the diagnosis would probably have reduced the number of subjects with diabetes detected by OGTT [32]. HbA_1c_ is known to be affected by anaemia. Hb was measured in the Tromsø Study 2007-2008, but not simultaneously as OGTT. However, anaemia is not a source of error when analysing HbA_1c_ with the HPLC method used in our study as the analysis is not performed if there are too few or too many erythrocytes in the sample. Haemolytic anaemia could result in falsely low HbA_1c_, but the condition is rare in our population and is not likely to affect the results. Other shortcomings are that we did not have information about retinopathy or other end organ diseases, and that we did not differentiate between type 1 and type 2 diabetes. However, as subjects in our study did not have previously diagnosed diabetes and age ranged from 30–87 years, most diabetes cases were likely to be type 2 diabetes. The cross-sectional study design is a major limitation when evaluating the impact of using different diagnostic criteria for diabetes. Prospective studies are needed to clarify which test detects the population with the highest risk of disease progression and complications of diabetes. 

## 5. Conclusions

The current HbA_1c_ and glucose criteria for diabetes defined different subjects with only modest overlap. Among those with diabetes detected by OGTT (regardless of HbA_1c_), isolated raised 2-hour plasma glucose was more common in subjects aged ≥ 60 years as compared to younger subjects, and in elderly women as compared to elderly men. As race, age, sex, and possibly BMI seem to affect HbA_1c_, FPG, and 2hPG and the relationship between these, creating an algorithm for choice of diagnostic test in different subgroups is a possibility and may be beneficial. If the aim is to detect as many patients with diabetes as possible, our data suggest that OGTT would be preferable for those aged ≥ 60 years, and especially women, while HbA_1c_ would be preferable for the younger and those with low BMI. However, in order to decide which diagnostic test should be preferred, and whether race, age, sex, and/or BMI specific guidelines should be considered, prospective studies with micro- and macrovascular end-points are needed.

## Figures and Tables

**Table 1:** Number of participants planned to participate, invited to participate, attended, and completed OGTT in the Tromsø OGTT Study.

HbA_1c_ level in the sixth Tromsø	Number of subjects			
study survey (2007-2008)	Planned to participate	Invited to participate	Attended OGTT	Completed OGTT
5.3%	200	309	180	176
5.4%	200	308	195	194
5.5%	100	144	109	107
5.6%	100	164	128	123
5.7%	100	157	115	112
5.8–6.9%	All	3311	2793	2764

Total		4393	3520	3476

Abbreviations: Haemoglobin A_1c_, HbA_1c_; oral glucose tolerance test, OGTT.

The table summarises how many subjects were planned to participate in the OGTT Study, how many were invited to OGTT, how many attended, and how many who completed OGTT at different HbA_1c_ levels and in total.

**Table 2:** Diabetes detected by HbA_1c_ only, OGTT only and both, and by OGTT components (FPG and isolated 2hPG), by subgroups in the Tromsø OGTT Study.

Category	Subcategory	Subjects without diabetes	All subjects with diabetes *N* (% of total)	Subjects with diabetes detected by			Subjects with diabetes detected by OGTT regardless of HbA1c	
				HbA_1c_ only	OGTT only	Both HbA_1c_ and OGTT	Raised FPG (regardless of 2hPG)	Isolated raised 2hPG
				*N* (% of diabetes)	*N* (% of diabetes)	*N* (% of diabetes)	*N* (% of diabetes by OGTT)	*N* (% of diabetes by OGTT)
All		3182	294 (8.5)	95 (32.3)	130 (44.2)	69 (23.5)	119 (59.8)	80 (40.2)
Sex^†^	Men	1593	163 (9.3)	53 (32.5)	72 (44.2)	38 (23.3)	76 (69.1)	34 (30.9)
	Women	1589	131 (7.6)	42 (32.1)	58 (44.3)	31 (23.7)	43 (48.3)	46 (51.7)
Age (years)^†^	<60	1153	61 (5.0)	26 (42.6)	20 (32.8)	15 (24.6)	31 (88.6)	4 (11.4)
	≥60	2029	233 (10.3)	69 (29.6)	110 (47.2)	54 (23.2)	88 (53.7)	76 (46.3)
BMI (kg/m^2^)*	<25	865	47 (5.2)	25 (53.2)	17 (36.2)	5 (10.6)	9 (40.9)	13 (59.1)
	25–29	1491	121 (7.5)	33 (27.3)	56 (46.3)	32 (26.4)	54 (61.4)	34 (38.6)
	≥30	824	124 (13.1)	37 (29.8)	56 (45.2)	31 (25.0)	55 (63.2)	32 (36.8)
Smoking status	Smoker	746	69 (8.5)	27 (39.1)	25 (36.3)	17 (24.6)	23 (54.8)	19 (45.2)
	Nonsmoker	2436	225 (8.5)	68 (30.2)	105 (46.7)	52 (23.1)	96 (61.1)	61 (38.9)
PAS tertile*	Low	949	121 (11.3)	26 (21.5)	63 (52.1)	32 (26.4)	50 (52.6)	45 (47.4)
	Medium	1079	101 (8.6)	39 (38.6)	38 (37.6)	24 (23.8)	39 (62.9)	23 (37.1)
	High	1154	72 (5.9)	30 (41.7)	29 (40.3)	13 (18.1)	30 (71.4)	12 (28.6)
TG (mmol/L)*	<1.2	1855	115 (5.8)	52 (45.2)	46 (40.0)	17 (14.8)	37 (58.7)	26 (41.3)
	1.2–2.6	1180	150 (11.3)	39 (26.0)	66 (44.0)	45 (30.0)	70 (63.1)	41 (36.9)
	>2.6	146	28 (16.1)	3 (10.7)	18 (64.3)	7 (25.0)	12 (48.0)	13 (52.0)

Data are *N* (%). Pearson Chi-square test was used for subgroup analysis. ^∗^ *P* < 0.05 for subjects with diabetes detected by HbA_1c_ only as compared to OGTT only. ^†^ *P* < 0.05 for subjects with raised FPG as compared to isolated raised 2hPG.

Abbreviations: Haemoglobin A_1c_: HbA_1c_: oral glucose tolerance test: OGTT; fasting plasma glucose: FPG; 2-hour plasma glucose: 2hPG; physical activity score: PAS; triglycerides: TG.

**Table 3:** Characteristics of subjects with diabetes detected by OGTT only, HbA_1c_ only, and both, and by OGTT components (FPG and isolated 2hPG) in the Tromsø OGTT Study.

	Subjects without diabetes	All subjects with diabetes	Subjects with diabetes detected by			Subjects with diabetes detected by OGTT regardless of HbA_1c_	
			HbA_1c_ only	OGTT only	Both HbA_1c_ and OGTT	Raised FPG (regardless of 2hPG)	Isolated raised 2hPG
*N*	3182	294	95	130	69	119	80
Women (%)	49.9	44.6	44.2	44.6	44.9	36.1^†^	57.5
Age (years)	60.7 ± 10.3	64.5 ± 8.6	63.7 ± 10.0	64.7 ± 7.4	65.3 ± 8.8	64.0 ± 8.6^†^	66.3 ± 6.6
BMI (kg/m^2^)	27.7 ± 4.3	29.7 ± 5.2	29.2 ± 6.0	29.5 ± 4.5	30.9 ± 5.1	30.6 ± 4.9^†^	29.1 ± 4.4
Smokers (%)	23.4	23.5	28.4	19.2	24.6	19.3	23.8
SBP (mmHg)	139 ± 22	147 ± 24	140 ± 22*	150 ± 22	151 ± 28	150 ± 25	151 ± 23
PAS (hours/week)	0.94 (0.0, 4.5)	0.38 (0.0, 4.5)	0.94 (0.0, 4.5)*	0.38 (0.0, 4.5)	0.38 (0.0, 3.0)	0.38 (0.0, 4.5)	0.19 (0.0, 4.5)
HbA_1c_ (%)	5.9 ± 0.3	6.4 ± 0.3	6.6 ± 0.1*	6.1 ± 0.2	6.7 ± 0.3	6.4 ± 0.4^†^	6.2 ± 0.3
FPG (mmol/L)	5.5 ± 0.5	6.6 ± 0.96	6.0 ± 0.6*	6.7 ± 0.9	7.4 ± 0.9	7.5 ± 0.7^†^	6.1 ± 0.6
2hPG (mmol/L)	5.6 ± 1.7	9.8 ± 3.3	7.0 ± 2.1*	11.1 ± 2.8	11.2 ± 3.3	10.2 ± 3.4^†^	12.5 ± 1.3
HOMA-IR	2.18 ± 1.65	4.18 ± 3.56	3.38 ± 2.79*	4.34 ± 4.05	4.96 ± 3.31	5.34 ± 4.51^†^	3.38 ± 1.97
QUICKI	0.35 ± 0.04	0.33 ± 0.04	0.34 ± 0.06*	0.32 ± 0.03	0.31 ± 0.03	0.31 ± 0.03^†^	0.33 ± 0.04
ISI_0.120_	4.77 ± 1.25	4.01 ± 1.27	4.42 ± 1.61*	3.87 ± 1.07	3.71 ± 0.92	3.72 ± 0.94	3.96 ± 1.12
TG (mmol/L)	1.32 ± 0.81	1.64 ± 0.94	1.34 ± 0.59*	1.80 ± 1.16	1.78 ± 0.75	1.76 ± 0.90	1.84 ± 1.21

Data are means ± SD or median (5, 95 percentile). Logistic regression was used for categorical variables and univariate analysis of variance with Bonferroni post-hoc adjustment or Mann-Whitney *U* test for continuous variables. ^∗^ *P* < 0.05 as compared to OGTT only. ^†^ *P* < 0.05 as compared to isolated raised 2hPG. Abbreviations: Haemoglobin A_1c_: HbA_1c_; oral glucose tolerance test, OGTT; fasting plasma glucose, FPG; 2-hour plasma glucose: 2hPG; systolic blood pressure: SBP; physical activity score: PAS; homeostasis model assessment-insulin resistance: HOMA-IR; quantitative insulin-sensitivity check index: QUICKI; insulin sensitivity index, ISI_0.120_; triglycerides, TG.

## Abbreviations

Hb: Haemoglobin
HbA_1c_: Haemoglobin A_1c_
OGTT: Oral glucose tolerance test
FPG: Fasting plasma glucose
2hPG: 2-hour plasma glucose
HPLC: High precision liquid chromatography
BMI: Body mass index
HOMA-IR: Homeostasis model assessment-insulin resistance
QUICKI: Quantitative insulin-sensitivity check index
ISI_0.120_: Insulin sensitivity index,
PAS: Physical activity score
TG: Triglycerides.
